# Supplementary material for: Global observations of reflectors in the mid-mantle with implications for mantle structure and dynamics
Source: Nat Commun. 2018 Jan 26;9:385. doi: 10.1038/s41467-017-02709-4 (PMC5786065; doi:10.1038/s41467-017-02709-4)
Supplement: Supplementary file 1 — Supplementary Information [file 41467_2017_2709_MOESM1_ESM.pdf]

Supplementary Information:

Global Observations of Reflectors in the Mid-Mantle with Implications for Mantle Structure and Dynamics

Lauren Waszek, Nicholas C. Schmerr, Maxim D. Ballmer

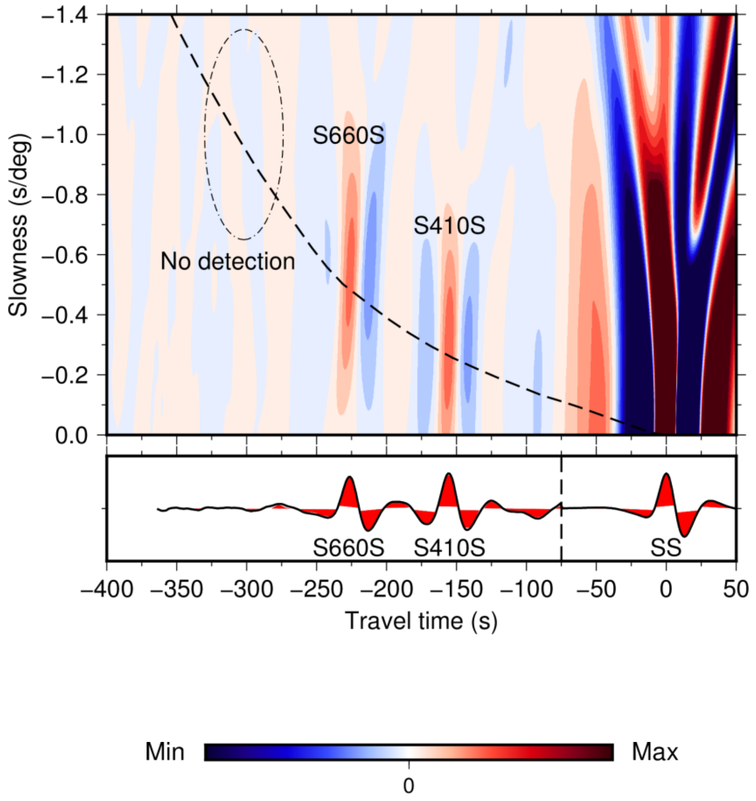

Supplementary Figure 1. Global stack of all of the data.

Areas of colour are arrivals of seismic energy. Precursors from the 410 and 660 km discontinuities are indicated. The cross-section underneath is taken through the predicted arrival time and slowness of SS precursors (dashed line). Bootstrap resampling is used to estimate 95% confidence levels of the cross-section. Red peaks

are defined as significant. The 410 and 660 km discontinuities are indicated. No energy is observed from mid-mantle depths, confirming the lack of a global discontinuity.

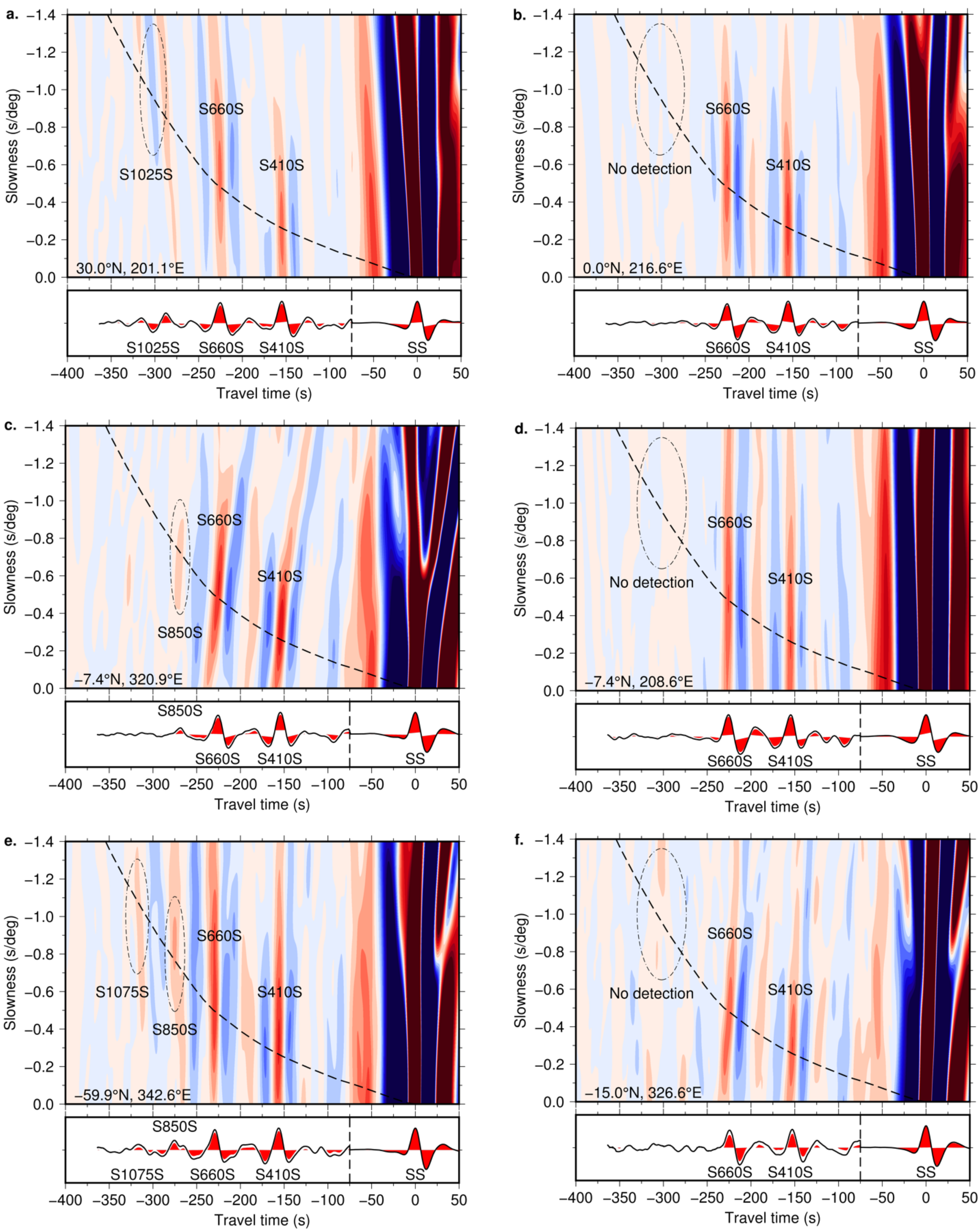

Supplementary Figure 2. Stacks for 15°, 7.5° and 5° caps.

High quality vespagrams for (a,b) 15° caps, (c,d) 7.5° caps, (e,f) 5° caps. An observed reflector and a non-detection is shown for each.

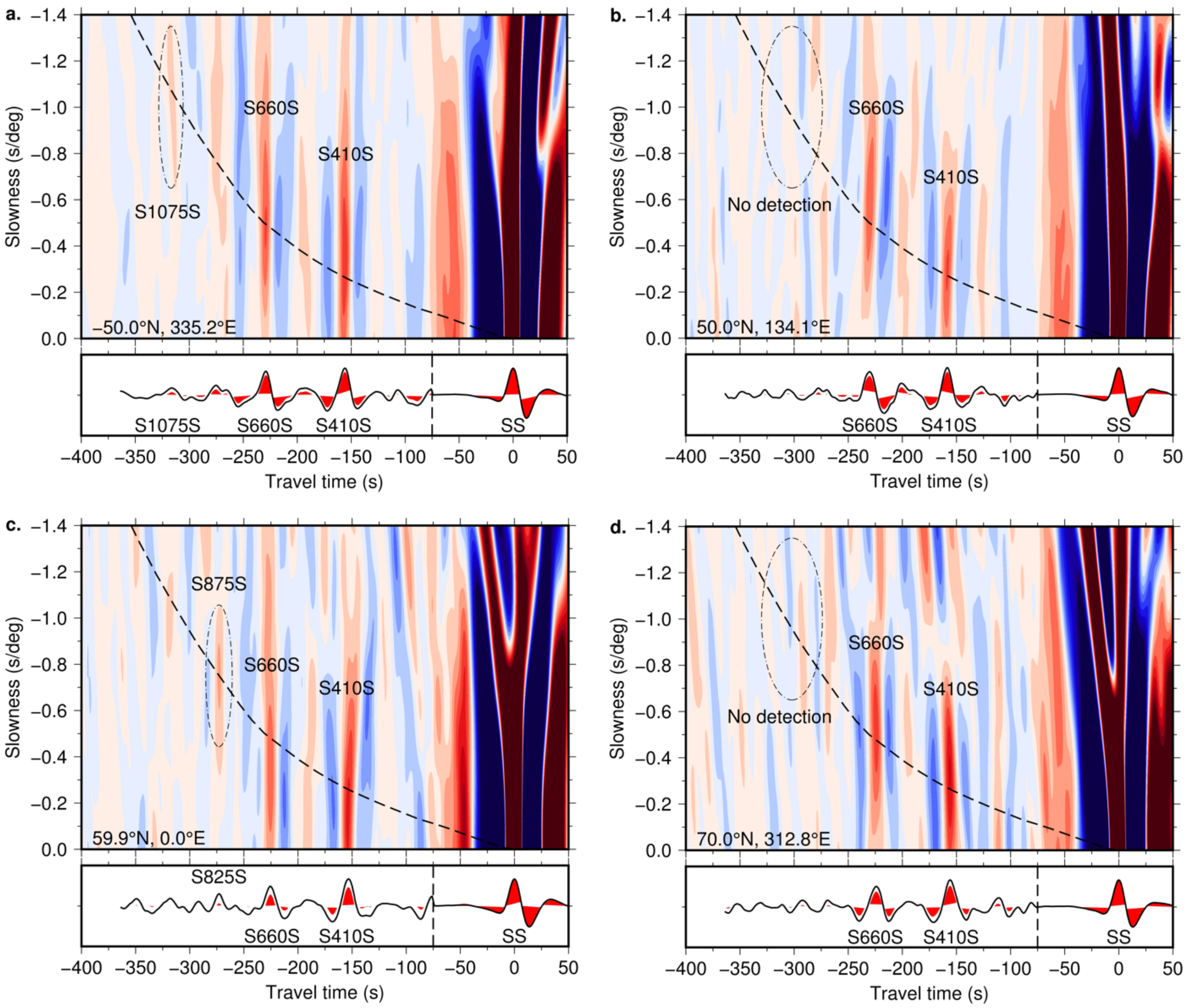

Supplementary Figure 3. Stacks for 10° caps for quality B and C data.

Vespagrams for 10° caps, for (a,b) B quality data, and (c,d) C quality data. An observed reflector and a non-detection is shown for each. The B quality data is characterised by additional arrivals than in the A quality data, which primary arrive away from the theoretical travel time and slowness of the precursors. The C quality data is noisier still, with small, non-significant arrivals throughout the vespagram. In all vespagrams, the observed precursors are significant (red peaks in the cross-section), and regions of non-detection all correspond to cross-sections with no significant peaks.

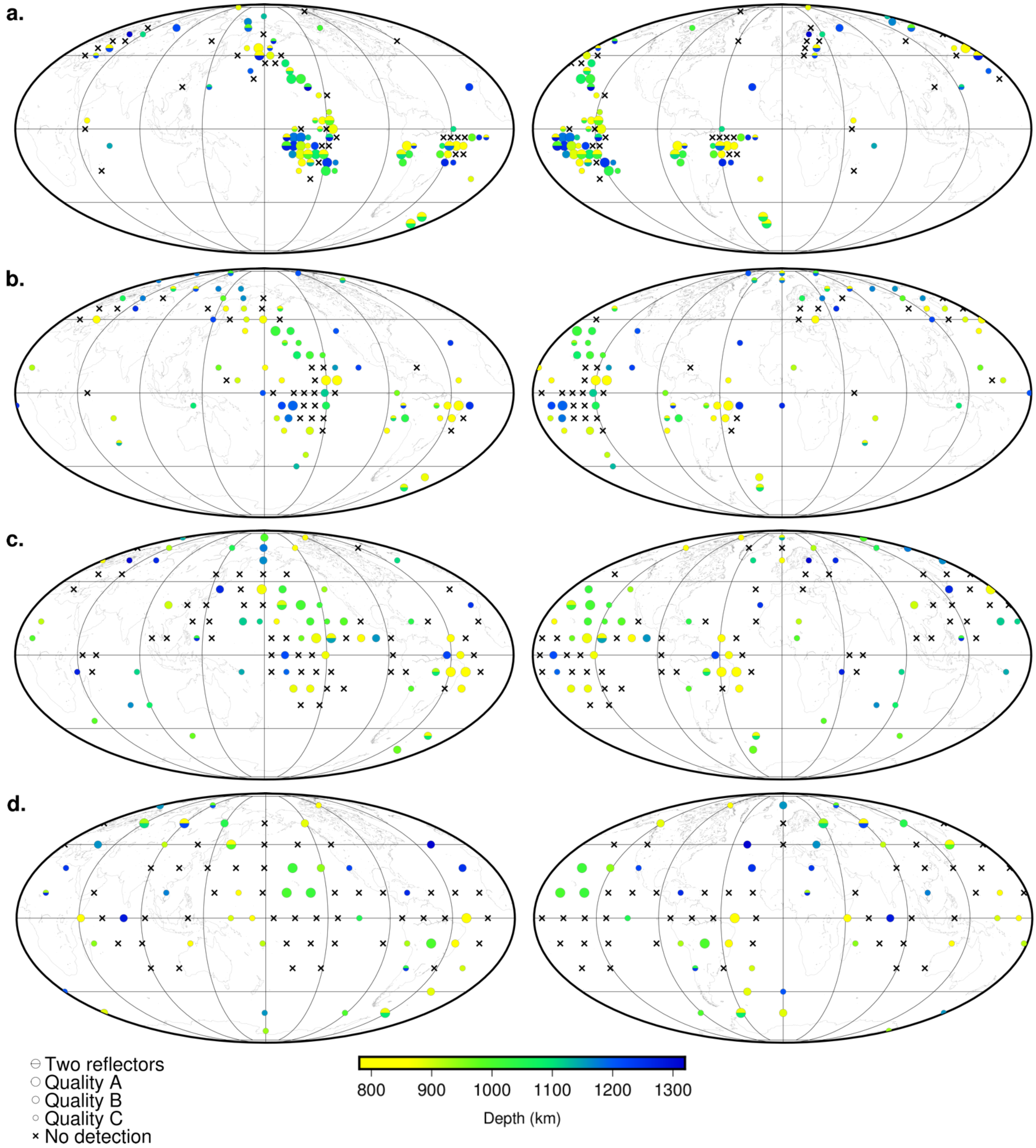

Supplementary Figure 4. Depths of reflectors for all cap sizes.

a. 5° caps. **b**. 7.5° caps. **c**. 10° caps. **d**. 15° caps.

Maps showing detections and depths of reflectors for different cap sizes.

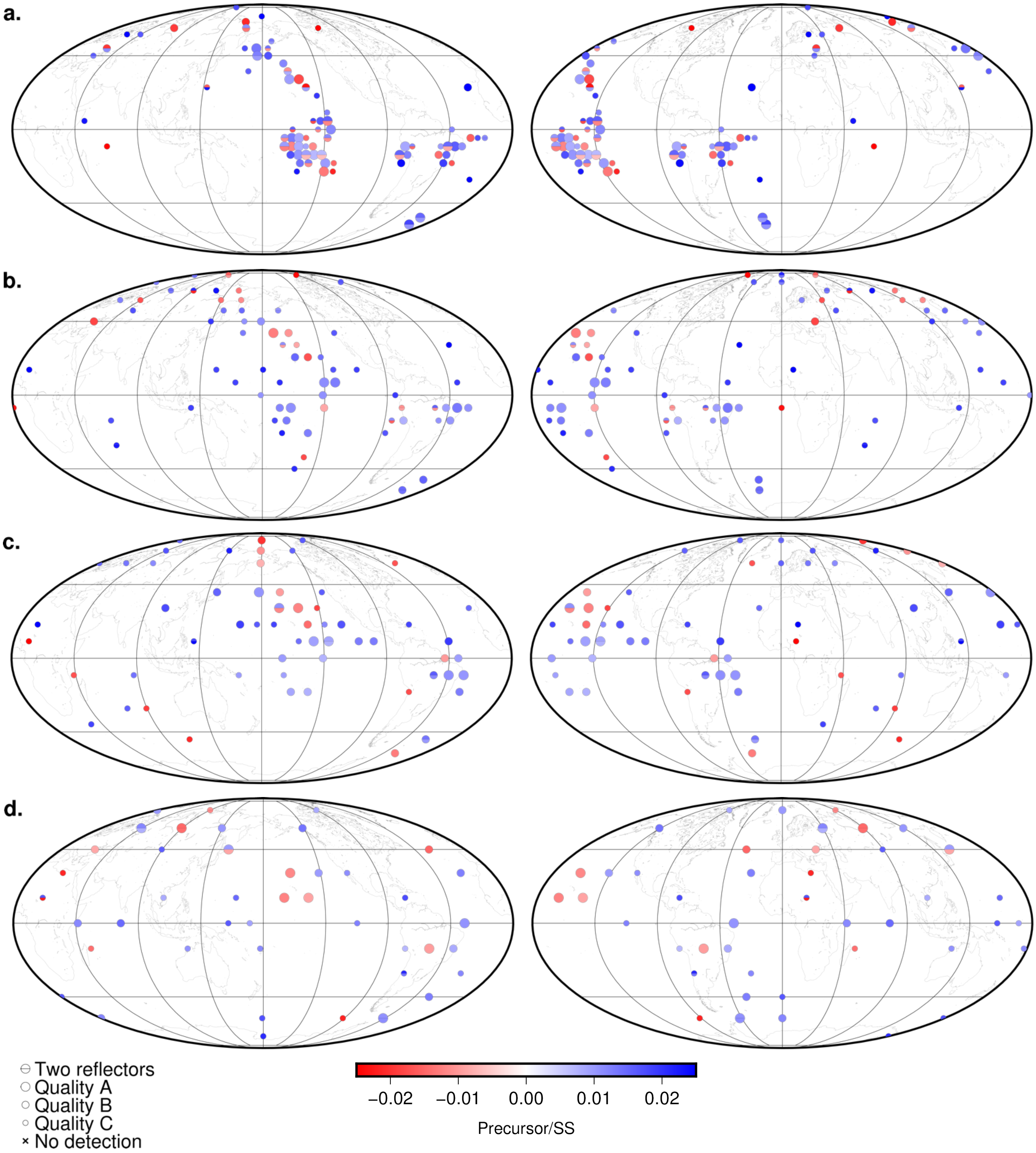

Supplementary Figure 5. Precursor/SS amplitude ratios of reflectors for all cap sizes.

a. 5° caps. **b**. 7.5° caps. **c**. 10° caps. **d**. 15° caps.

Maps showing amplitude ratios of discontinuities for different cap sizes.

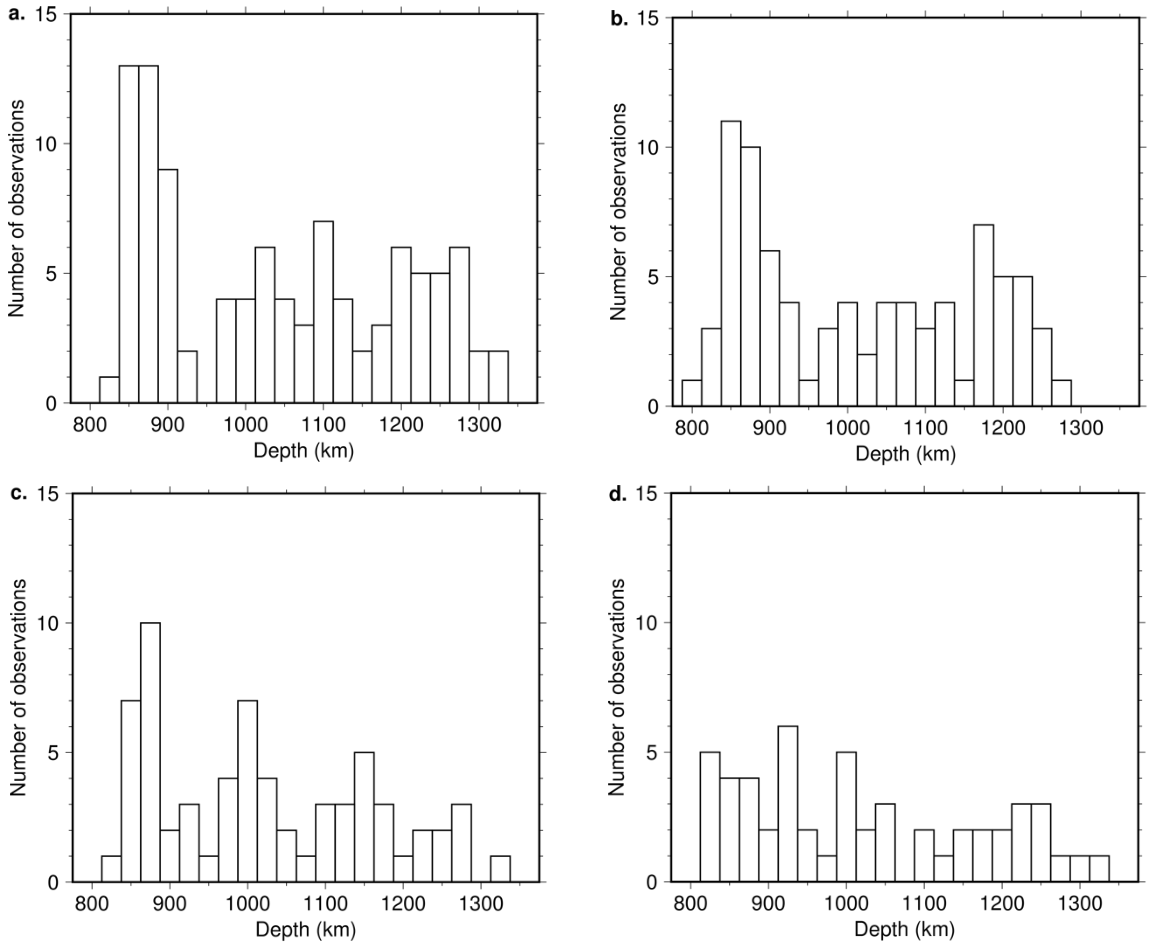

Supplementary Figure 6. Depths of reflectors for all cap sizes.

a. 5° caps. **b**. 7.5° caps. **c**. 10° caps. **d**. 15° caps.

Histograms showing depths of reflectors for all cap sizes.

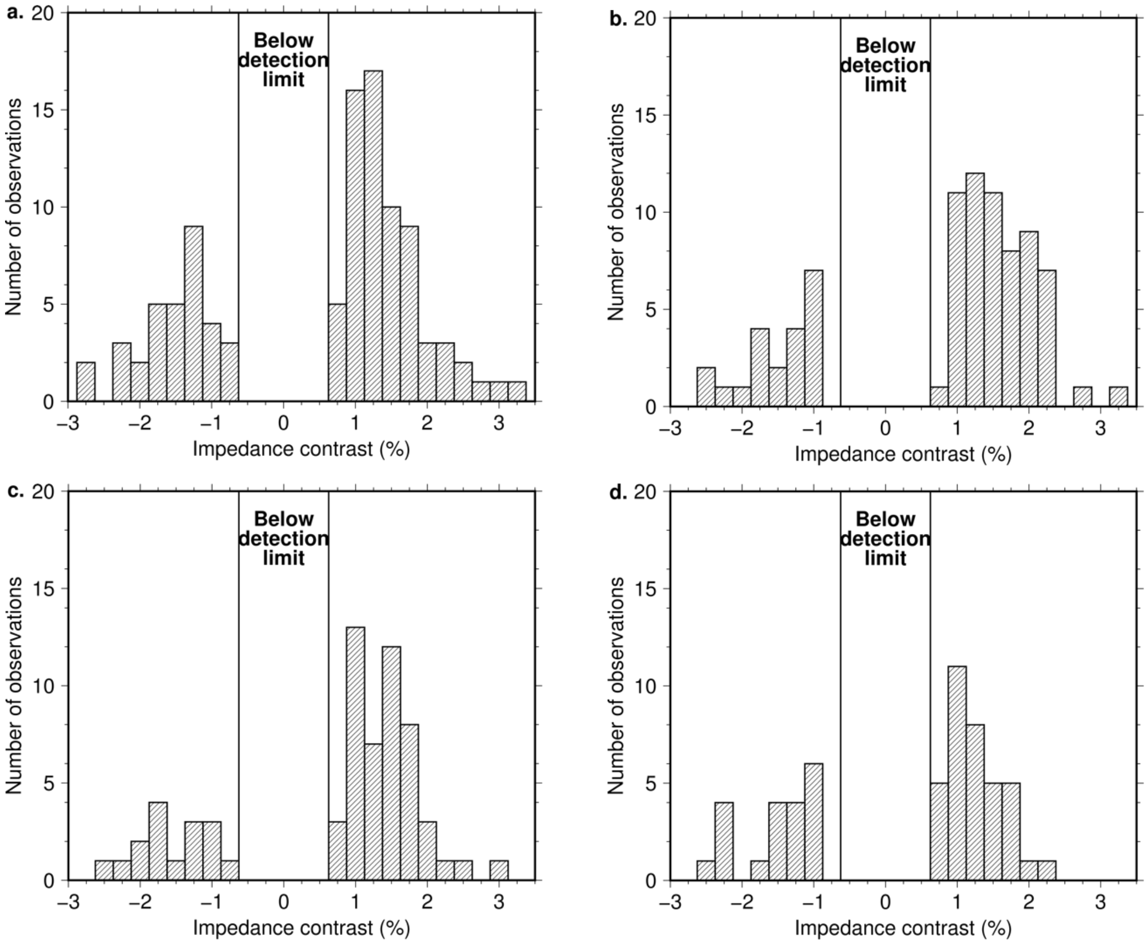

Supplementary Figure 7. Impedance contrasts of observed mid-mantle reflectors for all cap sizes.

a. 5° caps. **b**. 7.5° caps. **c**. 10° caps. **d**. 15° caps.

Histogram showing impedance contrasts estimated from shear wave velocity contrasts of discontinuities for all cap sizes. Impedance contrasts below the detection limit are indicated.

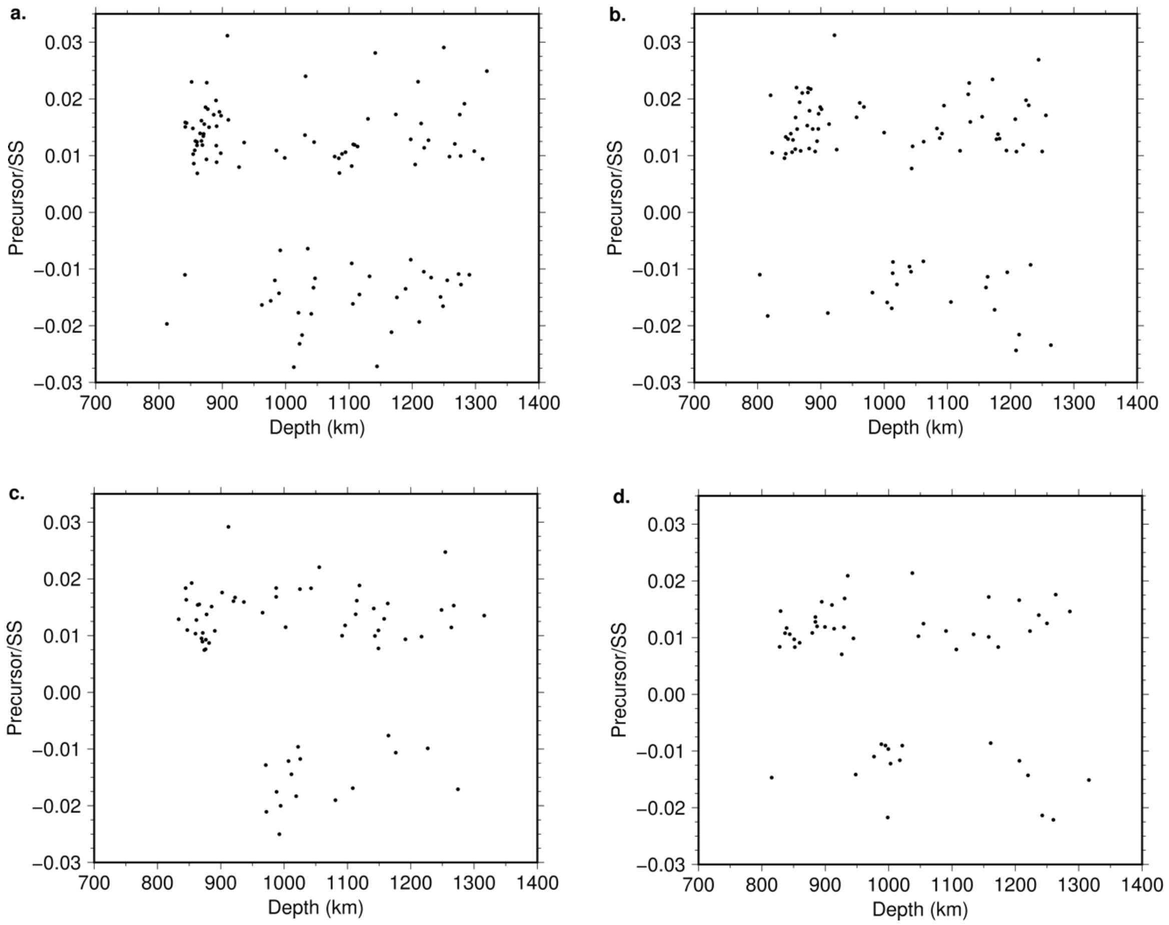

Supplementary Figure 8. Amplitude ratios as a function of reflector depth for all cap sizes.

a. 5° caps. **b**. 7.5° caps. **c**. 10° caps. **d**. 15° caps.

Plots showing the lack of relationship between observed depth of reflectors and their amplitude of their respective precursors with respect to SS.

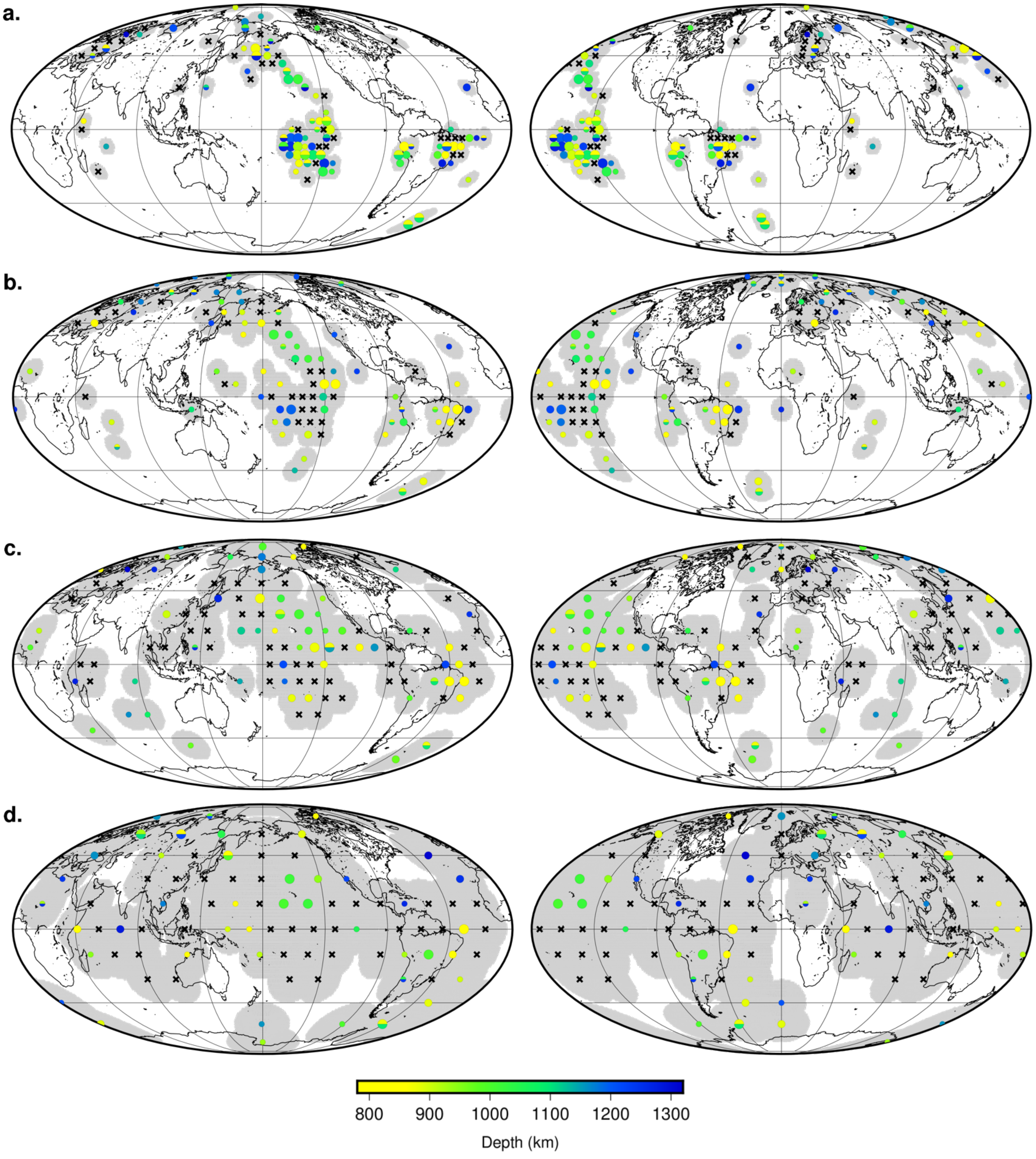

Supplementary Figure 9. Data coverage for all cap sizes.

a. 5° caps. **b**. 7.5° caps. **c**. 10° caps. **d**. 15° caps.

Shaded areas correspond to regions included in bins. Non-shaded areas are regions with

insufficient data coverage, or poor quality stacks that were removed after quality checking.

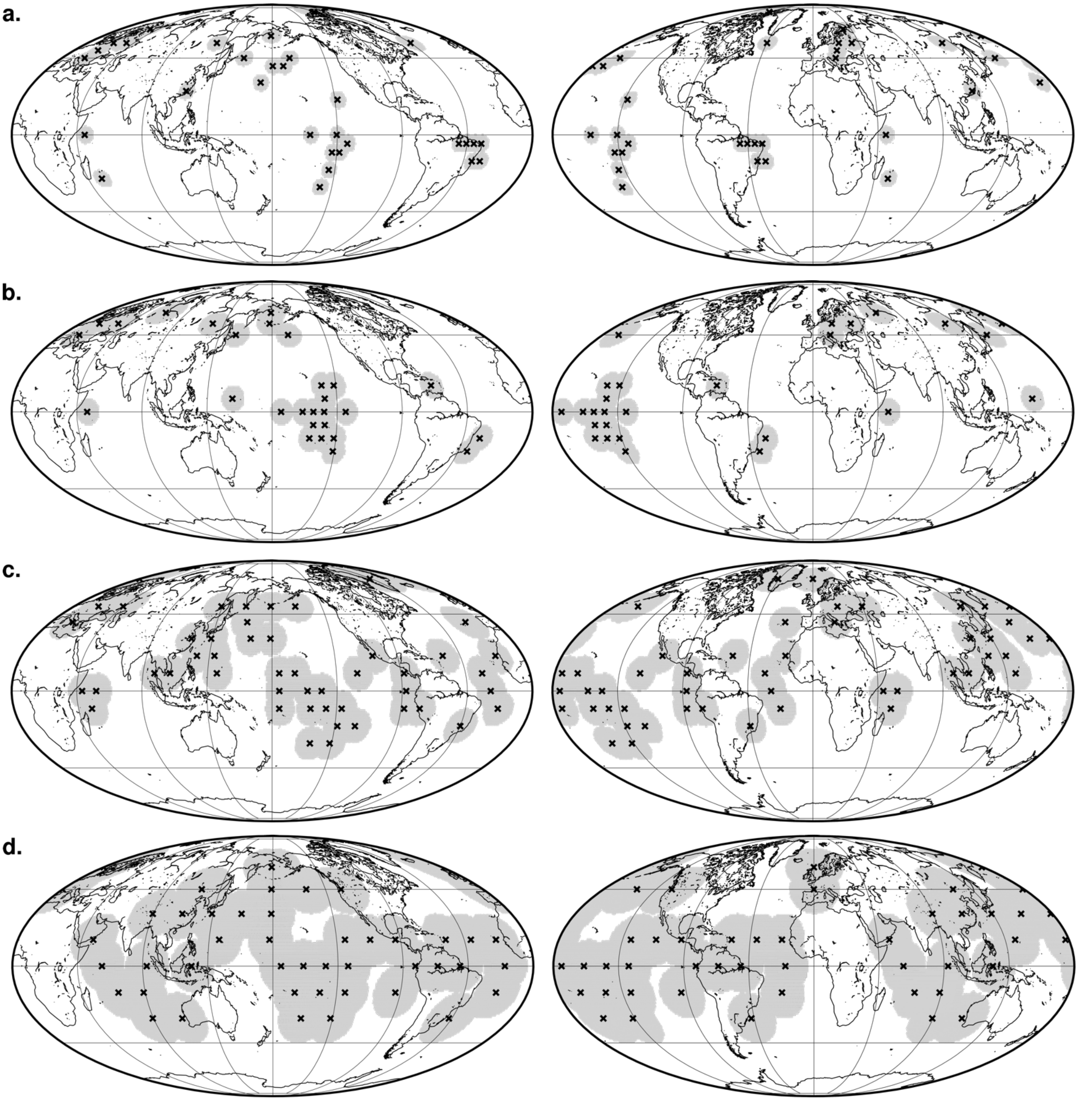

Supplementary Figure 10. Data coverage for non-observations for all cap sizes (Figure S5).

a. 5° caps. **b**. 7.5° caps. **c**. 10° caps. **d**. 15° caps.

Shaded areas correspond to regions included in bins. Non-shaded areas are regions with

insufficient data coverage, or poor quality stacks that were removed after quality checking.

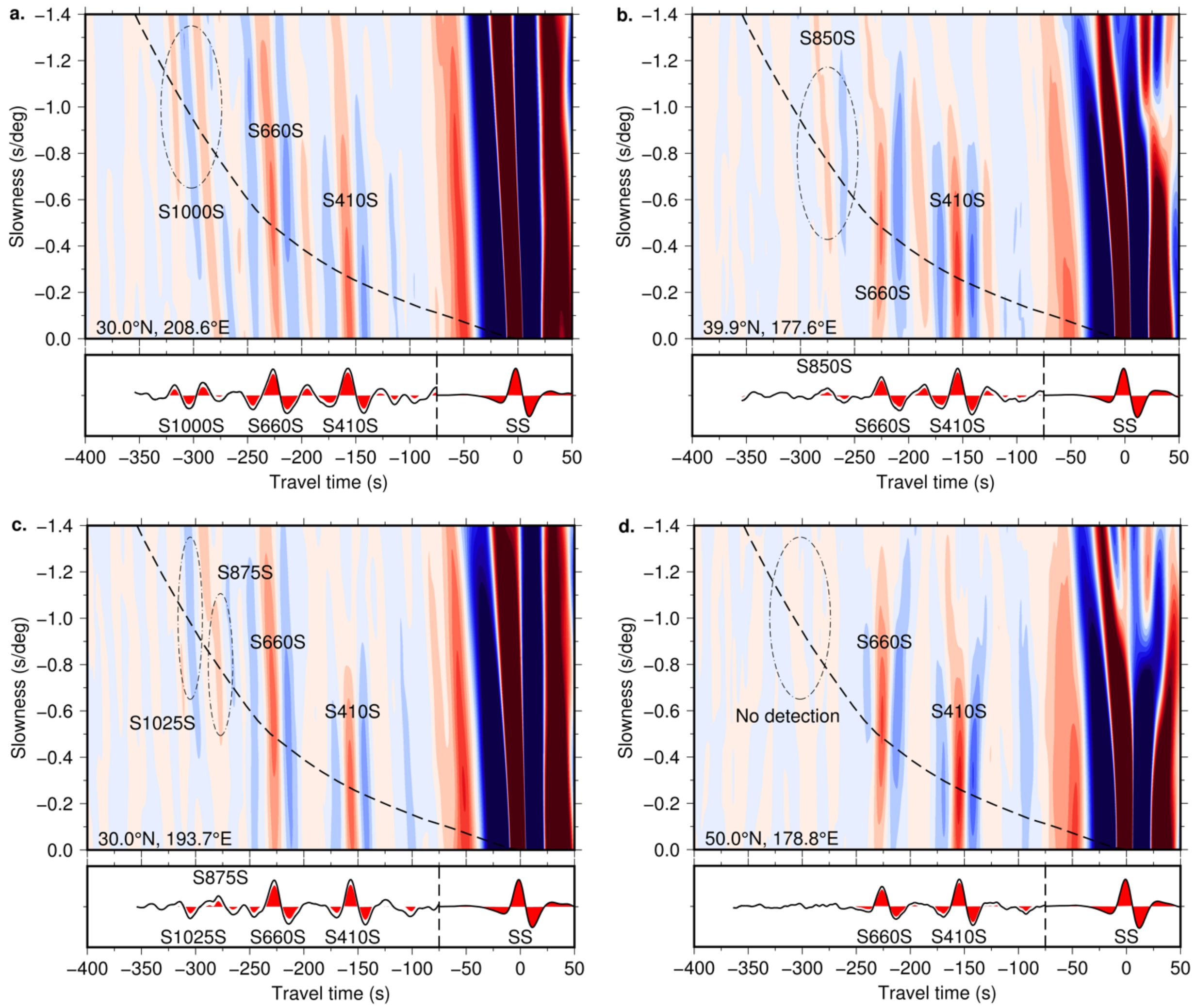

Supplementary Figure 11. Vespagrams for 10° caps corrected with S20RTS.

a. Negative polarity reflector (number of records, NR=1492). **b**. Positive polarity reflector (NR=1486). **c**. Multiple mid-mantle reflectors (NR=1348). **d**. No mid-mantle reflectors (NR=1647), a “non-detection”.

The data are corrected prior to stacking using S20RTS travel time delays calculated for a discontinuity at 1000 km depth. Limited difference is observed to the uncorrected data, likely due to averaging over the regional structure.

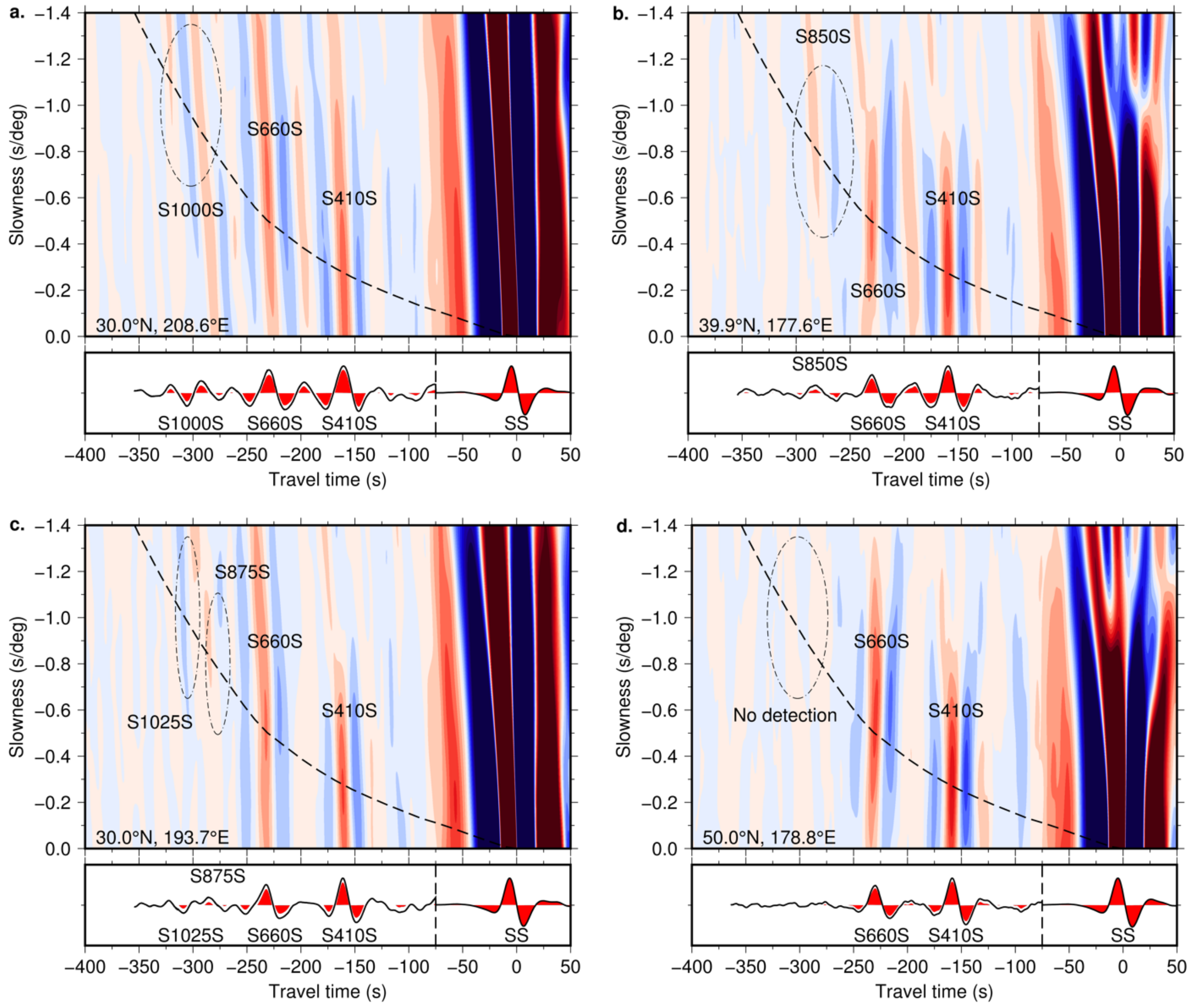

Supplementary Figure 12. Vespagrams for 10° caps corrected with SEMUCB-WM1.

a. Negative polarity reflector (number of records, NR=1492). **b**. Positive polarity reflector (NR=1486). **c**. Multiple mid-mantle reflectors (NR=1348). **d**. No mid-mantle reflectors (NR=1647), a "non-detection".

The data are corrected prior to stacking using SEMUCB-WM1 travel time delays calculated for a discontinuity at 1000 km depth. Limited difference is observed compared to the uncorrected data, however some variations in waveform are detected.

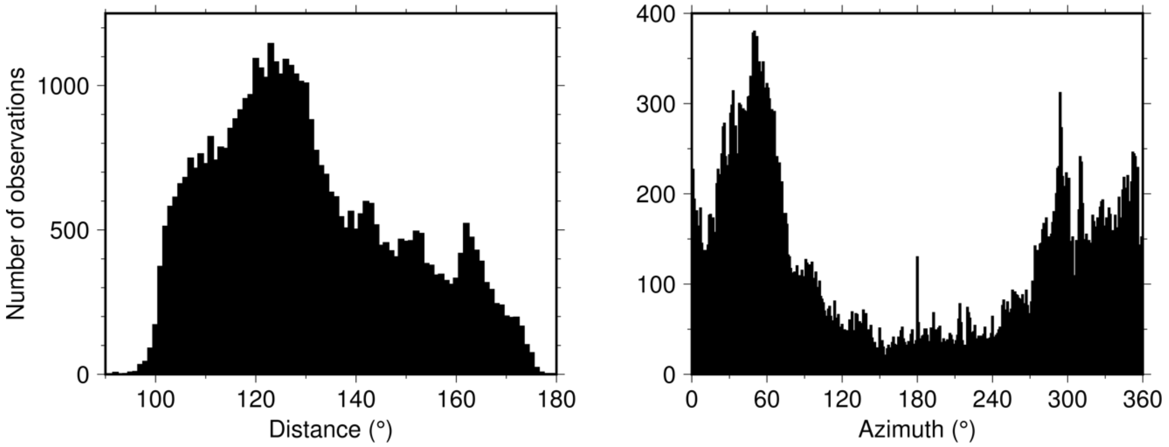

Supplementary Figure 13. Distance and azimuthal distribution of the data.

Data were selected in the distance range from approximately 100 to 180°, with no constraints on azimuth. The plots show a good distribution of data across all epicentral distances. The spread of azimuths is good on a global scale, revealing the future potential for investigations of observations as a function of direction in some regions.

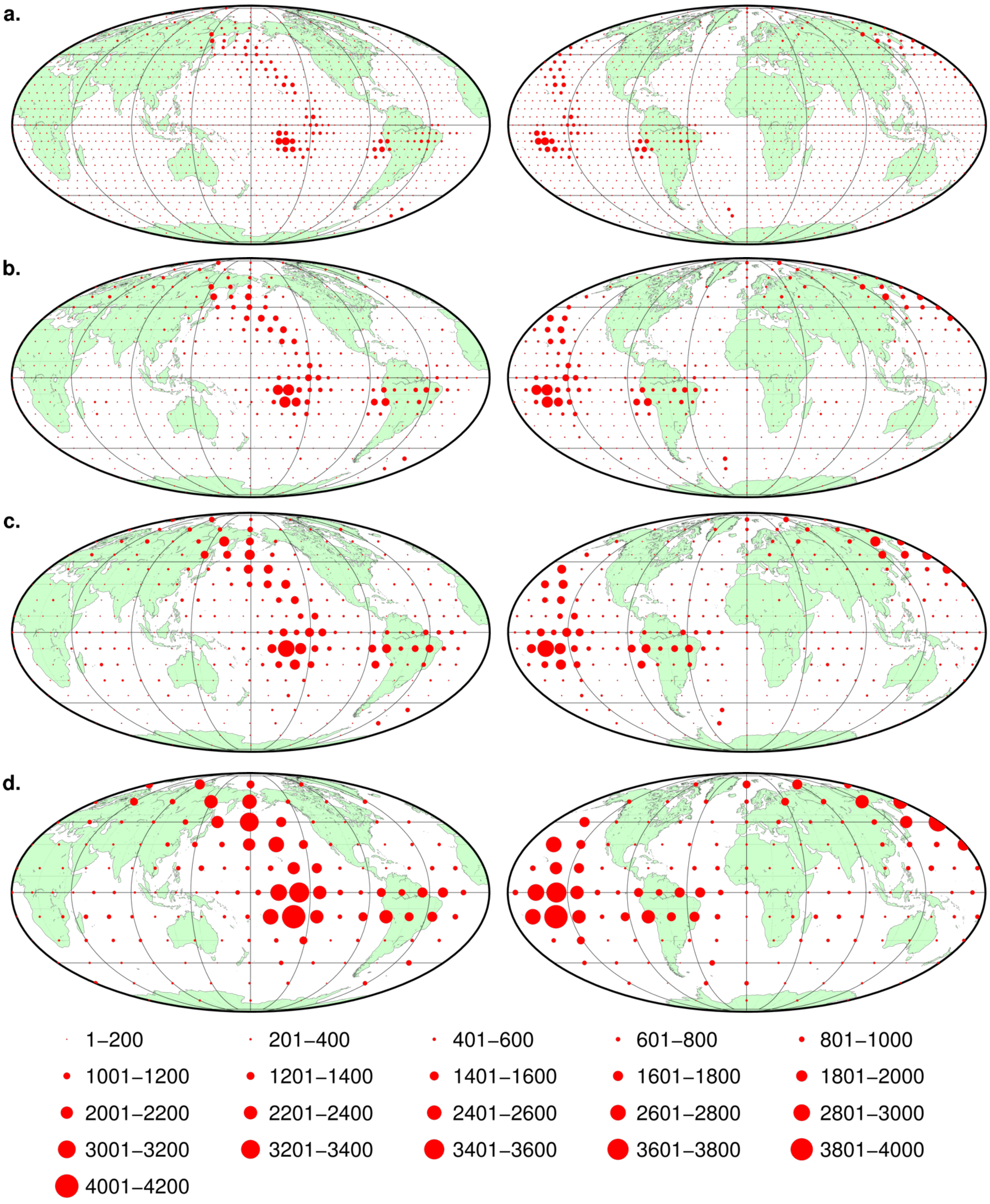

Supplementary Figure 14. Data distribution for all cap sizes.

a. 5° caps. **b**. 7.5° caps. **c**. 10° caps. **d**. 15° caps.

The size of circles in each bin shows the number of data in each bin, partitioned into increments of 200 data points as per the key. Only bins which were not stacked due to insufficient data are not shown; bins discarded due to low quality are included here.

Cap size	Domains		Observation p-value	Positive polarity p-value
Combined	Fast	Neutral	**0.034**	0.316
	Neutral	Slow	**0.013**	0.140
	Slow	Fast	**0.001**	**0.099**
5° caps	Fast	Neutral	0.331	0.166
	Neutral	Slow	**0.003**	0.104
	Slow	Fast	**0.003**	**0.034**
7.5° caps	Fast	Neutral	0.341	0.179
	Neutral	Slow	**0.089**	0.221
	Slow	Fast	**0.072**	**0.099**
10° caps	Fast	Neutral	**0.076**	0.192
	Neutral	Slow	0.356	0.429
	Slow	Fast	0.251	0.227
15° caps	Fast	Neutral	0.233	0.117
	Neutral	Slow	0.500	0.433
	Slow	Fast	0.322	0.179

Supplementary Table 1. Results from statistical analyses comparing the observations and polarities of reflectors between two seismic domains.

A one-tail z-test was used to determine whether the observations from two seismic domains differ significantly from each other. Tests were performed for the proportion of reflectors, and proportion of reflectors with positive polarity; p-values of 0.1 or less are bold in the table.

Cap size	Domain	Fast	Neutral	Slow
	Global proportion (%)	23.2	69.9	6.9
Combined caps	Data coverage of domain (%)	68.6	68.8	67.4
	Proportional data coverage (%)	23.2	70.0	6.8
5° caps	Data coverage of domain (%)	10.7	13.7	20.7
	Proportional data coverage (%)	17.5	71.8	10.7
7.5° caps	Actual coverage of domain (%)	21.1	29.7	31.5
	Proportional data coverage (%)	17.6	74.7	7.8
10° caps	Actual coverage of domain (%)	44.5	51.5	56.8
	Proportional data coverage (%)	20.6	71.6	7.8
15° caps	Actual coverage of domain (%)	80.0	79.5	74.5
	Proportional data coverage (%)	23.4	70.1	6.5

Supplementary Table 2. Global distribution of domains and data coverage at mid-mantle depths as a percentage.

The percentage of each domain type globally is calculated from the cluster votes across the full depth range from 800 to 1300 km. The corresponding data coverage of our dataset for each domain type is calculated for each bin size (assigning domain type to each position and depth based on three votes or more), and the value shown represents the proportion of global coverage of that domain which is sampled. These values increase with bin size as expected. The proportional data coverage refers to the proportional sampling of each domain type for each bin size. Our proportional data

coverage corresponds closely to the actual geographical proportions of each type of domain.
